# Identification and Characterization of MicroRNAs Involving in Initial Sex Differentiation of *Chlamys farreri* Gonads

**DOI:** 10.3390/biology11030456

**Published:** 2022-03-16

**Authors:** Xixi Li, Siyu Lin, Shutong Fan, Xiaoting Huang, Zhifeng Zhang, Zhenkui Qin

**Affiliations:** 1 Ministry of Education Key Laboratory of Marine Genetics and Breeding, College of Marine Life Sciences, Ocean University of China, Qingdao 266003, China; lclixixi@163.com (X.L.); 15563886136@163.com (S.L.); fanshutong689@163.com (S.F.); xthuang@ouc.edu.cn (X.H.); 2 Key Laboratory of Tropical Aquatic Germplasm of Hainan Province, Sanya Oceanographic Institution, Ocean University of China, Sanya 572000, China

**Keywords:** microRNA, gonadal sex, initial sex differentiation, *Chlamys farreri*

## Abstract

**Simple Summary:**

Sex formation of gonads encompasses two ancient and highly conserved biological processes, sex determination and sex differentiation. The processes are strictly regulated by a complex of gene networks. There is increasing evidence that miRNAs play key roles in many biological processes. however, information is limited in their contribution to sex differentiation in animals. In the present study, we identified the novel miRNAs involved in sex-related genes regulation and explored the miRNA–mRNA networks underlying the posttranscriptional regulation during the initial sex differentiation in Zhikong scallop, *Chlamys farreri*. Our findings provide an important basis for studying the sex differentiation mechanisms, as well as developing sex control techniques in bivalves.

**Abstract:**

Research on expressional regulation of genes at the initial sex differentiation of gonads will help to elucidate the mechanisms of sex determination and differentiation in animals. However, information on initial sex differentiation of gonads is limited in bivalves. MicroRNAs (miRNAs) are a class of endogenous small noncoding RNAs that can regulate the target gene expression at the posttranscription level by degrading the mRNA or repressing the mRNA translation. In the present study, we investigated the small RNAs transcriptome using the testes and ovaries of Zhikong scallop Chlamys farreri juveniles with a shell height of 5.0 mm, a critical stage of initial sex differentiation of gonads. A total of 75 known mature miRNAs and 103 novel miRNAs were identified. By comparing the expression of miRNAs between the ovary and testis, 11 miRNAs were determined to be differentially expressed. GO annotations and KEGG analyses indicated that many putative target genes that matched to these differentially expressed miRNAs participated in the regulation of sex differentiation. Furthermore, two selected miRNAs, cfa-novel_miR65 and cfa-miR-87a-3p_1, were confirmed to downregulate expressions of *Foxl2* (a female-critical gene) and *Klf4* (a male-critical gene), respectively, using a dual-luciferase reporter analysis. Our findings provided new insights into the initial sex differentiation of gonads regulated by miRNAs in bivalves.

## 1. Introduction

Sex is an important trait in multicellular bisexual organisms. Sex formation goes through two ancient and highly conserved biological processes, sex determination and sex differentiation [1]. The process is regulated by a complex network, which can be considered to be initiated by a sex determining trigger mediating the expression of sex determination genes, and ultimately, the male or female phenotype of sex is established [2]. However, the regulatory networks remain poorly understood in many species.

MicroRNAs (miRNAs) are highly conserved single stranded noncoding RNAs (20–24 nucleotides), whose functions result in mRNA degradation or translational repression by binding to the untranslated region or the coding regions of target mRNAs [3,4,5]. Recently, an increasing number of expression profiles of miRNAs have been revealed and investigated to elucidate their regulatory mechanisms through acting on target genes in a wide range of species and being involved in several biological processes, including cell proliferation and differentiation, immune defense, and organ development [6,7]. The role of miRNAs in the regulation of sex determination and differentiation have been investigated in several species. For example, in *Mus musculus*, miR-124 has been revealed to determine the fate of developing ovarian cells by repressing the expression of *Sox9*, a key gene for normal development of testis in most vertebrates [8]. In *Bactrocera dorsalis*, miR-1-3p can directly suppress the gene expression of *Bdtra* and be involved in the sex determination during early embryonic development [9]. In *Caenorhabditis elegans*, miR-35-41 prevents the aberrant activation of male developmental programs by interacting with the upstream and downstream sequences of *her-1* gene in hermaphrodite embryos [10]. Thus, miRNAs are essential and important in sex determination and differentiation.

Molluscs are the second largest phylum of invertebrates after Arthropoda. As an important representative group of molluscs, bivalves exhibit a variety mode of reproduction strategies, including dioecism and hermaphrodite, as well as sex reversal. Currently, there is limited data on the sex differentiation and gonadal development regulated by miRNAs that have been conducted in bivalves, such as in *Crassostrea gigas*, *Crassostrea hongkongensis*, and *Hyriopsis cumingii* [11,12,13]. In *C. gigas*, three female-biased miRNAs (miR-183, miR-96a, and miR-279) and three male-biased miRNAs (miR-8, miR-29, and novel_miR167) were discovered to exhibit a sex exclusive expression pattern in either male or female gonads, and miR-96a was speculated to maintain the primary oocyte in the first meiotic division by inhibiting *SPO11* expression in *C. gigas* ovarian cells [11]. In *C. hongkongensis*, six miRNAs with the significant expression differences (male-biased miRNAs: miR-1990, miR-1986, miR-29, and miR-8; female-biased miRNAs: miR-263b and miR-279) have been identified, and their predicted target genes were analyzed to be the molecules involved in sex differentiation and gonadal function maintenance [12]. Nevertheless, the above studies were all conducted in the fully developed gonads of sexually mature bivalves, and no relevant data on sex-biased miRNAs expression in the juveniles at the critical stage of initial gonadal sex differentiation have been reported. This deficiency affects the understanding of the mechanism by which miRNA regulates gonadal initial sex differentiation in bivalves.

The Zhikong scallop, *Chlamys farreri*, an important commercial aquaculture bivalve in China, is a good experimental model for initial sex differentiation study based on its sex-stable characteristics [14]. Liu et al. [15] reported that gonadogenesis of *C. farreri* starts in the juvenile with a shell height of 5.0 mm, and the histological differences between testis and ovary are initially showed in gonads of juveniles with shell height of 9.0 mm. Furthermore, Li et al. [16] determined the initial differentiation of gonadal sex occurs in *C. farreri* juveniles with a shell height of at least 5.0 mm through the algorithms of LOG_10_(*Dmrt1*/*Foxl2*) and LOG_10_(*Klf4*/*Foxl2*). Therefore, in this study, we employed small RNA transcriptome analysis, obtained the small RNA data in the gonads of *C. farreri* juveniles with 5.0 mm shell height, and identified the differentially expressed miRNAs between the testis and ovary during the initial sex differentiation stage. In addition, the miRNA–mRNA interaction networks were analyzed to explore the posttranscriptional regulation of initial sex differentiation. The present study illustrated the potential regulatory roles of miRNAs in the initial differentiation of gonadal sex and will help to elucidate the mechanisms of sex differentiation in bivalves.

## 2. Materials and Methods

### 2.1. Animals and Sampling

Healthy *C. farreri* juveniles with shell height of 5.0 mm were collected from the Changdao Bay (Yantai, China) and reared in seawater for 24 h before sampling. The gonads were dissected carefully under a stereoscopic microscope (Figure S1) and were then immediately snap-frozen in liquid nitrogen and then stored in −80 ℃ for total RNA extraction.

### 2.2. RNA Extraction, Small RNA Library Construction, and Sequencing

Total RNAs of the juvenile gonads were extracted using the MicroElute^®^ Total RNA Kit (Omega, Norcross, GA, USA). The RNA integrity and quality were assessed by 1.2% agarose gel electrophoresis and the Agilent 2100 Bioanalyzer (Agilent Technologies, Santa Clara, CA, USA). A small amount of RNA from each individual were firstly used to identify the sex as described previously [17,18]. Briefly, the expressions of three sex-related key genes *Foxl2*, *Dmrt1*, and *Klf4* were examined, and LOG_10_(*Dmrt1*/*Foxl2*) as well as LOG_10_(*Klf4*/*Foxl2*) values were calculated to determine the gonadal sex. Values below 0 or exceeding 0.7/0.6 indicated male or female individuals, respectively. Then, three ovary or testis small RNA libraries were constructed as follows: Total RNAs of each sample were isolated and purified by 15% urea denaturing polyacrylamide gel electrophoresis (PAGE), and RNAs corresponding to 1830 nt in length were excised from the gel and recovered. Then, the 18–30 nt small RNAs were ligated to adenylated 3′ adapters annealing with unique barcodes, followed by the ligation of 5′ adapters. The small RNAs were subsequently transcribed into cDNA and amplified using PCR to enrich the cDNA fragments. The 100–120 bp PCR products were recovered by gel extraction and inspected with the Agilent Bioanalyzer 2100 system. The constructed small RNA libraries were sequenced with the BGISEQ-500 platform (BGI-TECH, Wuhan, China).

The small transcriptome data were deposited in the Sequence Read Archive (SRA) database as PRJNA798567.

### 2.3. Processing of Sequencing Data

The clean reads were obtained by removing adapter sequences and low-quality sequences, as well as poly-N containing sequences from raw reads (SOAPnuke 1.5.0 filtersRNA-Q 2-q-z 18; BGI-TECH, Wuhan, China). Then, the clean reads ranging from 17 to 35 nt were selected for mapping back to the *C. farreri* genome (SRA accession number: PRJNA185465) by Bowtie2 (http://bowtie-bio.sourceforge.net/index.shtml, accessed on 20 March 2021). To ensure every unique small RNA was only annotated once, the mapped small RNA reads were compared with the Rfam database (http://rfam.xfam.org/, accessed on 20 March 2021) to exclude rRNAs (ribosomal RNAs), tRNAs (transfer RNA), snRNAs (small nuclear RNA), snoRNAs (small nucleolar RNAs), and repeat sequences. The remaining small RNA reads were queried against the miRBase22.1 (https://www.mirbase.org/, accessed on 20 March 2021) to identify the known miRNAs. The novel miRNAs were predicted through exploring the secondary structure of miRNA precursor, dicer cleavage sites, and the minimum free energy by miRDeep2 (https://www.mdc-berlin.de/content/mirdeep2-documentation, accessed on 20 March 2021).

### 2.4. Analysis of Differentially Expressed miRNAs

The miRNAs expression levels were normalized by TPM (transcript per million), in which normalized expression = (mapped read count/ total read count) × 1,000,000. Differentially expressed miRNAs (DEMs) between the ovary and testis were identified using DEGseq (http://bioconductor.org/packages/stats/bioc/DEGSeq/, accessed on 20 March 2021) based on the negative binomial distribution. miRNAs with *p*-value ≤ 0.05 and |log2 (fold change)| ≥ 1 were defined as DEMs.

### 2.5. Prediction of Potential Target Genes of DEMs

To predict the targeting genes of DEMs, the annotation files of *C. farreri* genome were utilized to detect the miRNAs binding sites using miRanda (http://www.miranda.org/, accessed on 1 April 2021) and RNAhybrid (http://bibiserv.techfak.uni-bielefeld.de/rnahybrid/, accessed on 1 April 2021), and the outcome data were integrated based on overlaps. Gene Ontology (GO) classification and Kyoto Encyclopedia of Genes and Genomes (KEGG) enrichment analyses were utilized to investigate the biological functions and pathways related to these candidate target genes. The terms with *p*-value ≤ 0.05 were regarded as significantly enriched.

### 2.6. Verification of DEMs Using RT-qPCR

To validate the obtained DEMs, the miRNA expression patterns were quantified using RT-qPCR. Briefly, total RNA was reverse-transcribed using the Mir-X miRNA First-Strand Synthesis Kit (Takara, Dalian, China) following the manufacturer’s protocol. Each miRNA was amplified by a specific forward primer (Table S1) and a universal reverse primer (Takara, Dalian, China), and 5S rRNA was used as an internal normalization control [13]. The amplification was carried out in a 10 μL volume using the SYBR Green Master Mix (Takara, Dalian, China) on the Roche LightCycler 480 Real-Time PCR System (Roche, Basel, Switzerland). The detections were performed with three parallel replicates and three technical replicates. Data were analyzed using the Roche LightCycler 480 system software version 1.5 (Roche, Basel, Switzerland), and the 2^−ΔΔCt^ method was used to calculate the relative level of miRNA [19]. All RT-qPCR assays were validated in compliance with “the MIQE guidelines” [20].

### 2.7. Validation of miRNA–mRNA Interaction by Dual-Luciferase Reporter Assay

The cfa-novel_miR65 and the cfa-miR-87a-3p_1 with the minimum free energy that targeting *Foxl2* and *Klf4*, respectively, were selected to validation miRNA–mRNA interaction. The pmirGLO Dual-Luciferase miRNA Target Expression Vector (Promega, Madison, WI, USA) was linearized with restriction enzymes *Sac* I and *Xho* I (Takara, Dalian, China). The target gene fragments containing predicted miRNA binding sites, and 100 bp upstream and downstream flanking sequences were amplified by specific PCR primers (Table S2) and *C. farreri* juvenile gonad cDNAs. The PCR products were then double enzymes digested (*Sac* I and *Xho* I), and the gel-purified fragments were cloned into the linearized pmirGLO to construct a wild-type (WT) vector. The mutant type (MT) vectors of target genes were generated by one-step PCR using site specific mutagenesis. Point mutation primers were designed in term of the predicted miRNA-binding sites, and the consecutive six nucleotides mismatches were introduced into the seed sequences (Table S2), and miRNA mimics of cfa-novel_miR65 and cfa-miR-87a-3p_1, as well as the negative control (NC) mimics (sense: 5′-UUGUACUACACAAAAGUACUG-3′, antisense: 5′-UUAACAUGAUGUGUUUUCAUG-3′), were synthesized by Sangon Biotech (Shanghai, China).

For the luciferase reporter assay, HEK293T cells were utilized and seeded in 24-well plates for about 24 h. When cells grew to 90–95% confluence, 500 ng recombinant vectors of WT or MT, and 2 µL of either miRNA mimics or NC mimics were co-transfected using Lipofectamine™2000 Transfection Reagent (Thermo Fisher Scientific, Wilmington, NC, USA). Each assay was performed with three parallel replicates. Forty-eight hours after transfection, the luciferase activities were measured by the Dual-Luciferase reporter assay system (Promega, Madison, WI, USA). Briefly, culture medium was removed, and cells were rinsed with 1 mL phosphate buffered saline (PBS). Then 100 µL 1× Passive Lysis Buffer (PLB) was dispensed into each well and shaken gently on a shaking table at room temperature for 30 min. After that, 10 µL cell lysates, as well as 50 µL Luciferase Assay Reagent II (LAR II), were dispensed into each well for a measurement of firefly luciferase activity with a 2-s delay and a 10-s read. Finally, 50 µL Stop & Glo^®^ Reagent were added into each well, followed by a 2-s delay and a 10-s read for Renilla luciferase activity detection. The relative reporter activity was normalized to Renilla luciferase activity.

### 2.8. Statistical Analysis

All experiments data were presented as the mean ± SD from three samples and three technical replicates. Significant differences between means were tested using one-way analysis of variance (ANOVA), followed by Tukey’s HSD test (SPSS software version 22.0; SPSS Inc., Chicago, IL, USA), and the statistically significant difference was set at *p* < 0.05, and extremely significant difference was at *p* < 0.01, respectively.

## 3. Results

### 3.1. Overview of Small RNA Sequencing Statistics in C. farreri Gonads

**Figure 1:**
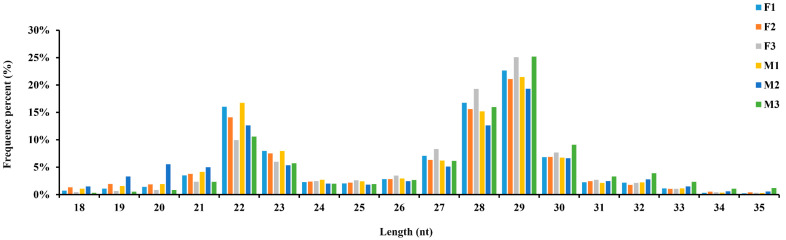
Length distribution of small RNA sequences from the ovary and testis libraries of *C. farreri*.

To identify the small RNAs that participating in the initial sex differentiation of *C. farreri* gonads, six libraries, including three (F1, F2, and F3) from ovaries of 5 mm juveniles and the other three (M1, M2, and M3) from testes, were constructed and sequenced. In total, 179,393,290 raw reads (6.2 Gb) were obtained. After removed adapter sequences, low-quality sequences, and containing poly-N sequences, 169,498,806 clean reads remained for further analysis (Table 1). The length distributions of the clean reads were similar between the ovary and testis (Figure 1), with a peak at 21–23 nt representing the typical size of miRNAs and the other peak ranged from 27–30 nt, representing piwi-interacting RNAs (piRNAs). A total of 127,540,441 clean reads were mapped to the *C. farreri* genome, and the mapping rate varied among libraries ranged from 68.19% to 79.02% (Table 1).

### 3.2. Identification of miRNAs

**Figure 2:**
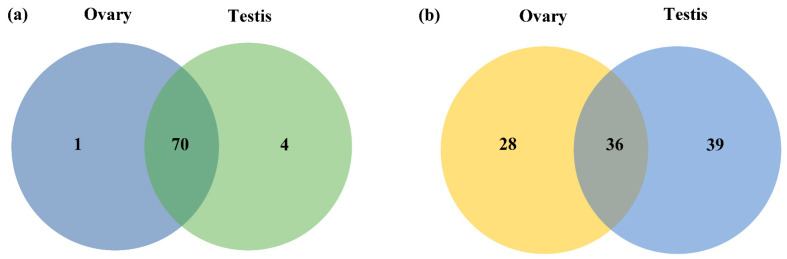
Overview of miRNAs identified from the transcriptome. (**a**) Distributions of known miRNAs between the ovary and the testis. (**b**) Distributions of novel miRNAs between the ovary and the testis.

**Figure 3:**
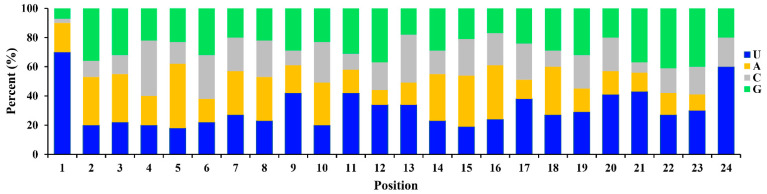
Nucleotide bias at each position in known mature miRNAs of *C. farreri*. Number of *x*-axis represents each position of miRNA.

The clean reads mapped to genome were used for miRNAs identification. A total of 75 known mature miRNAs were identified by aligned against the miRBase22.1 (Table S4), of which 70 known mature miRNAs were expressed in both ovaries and testes (Figure 2a). The nucleotide bias of each position in the known mature miRNAs were analyzed, and U, as well as A, were found to be dominant at the first position (Figure 3). The clean reads that were not annotated in database were polled for novel miRNAs prediction by miRDeep2. 103 novel miRNAs were identified (Table S5 and Figure 2b).

### 3.3. DEMs Identified between Ovary and Testis

**Figure 4:**
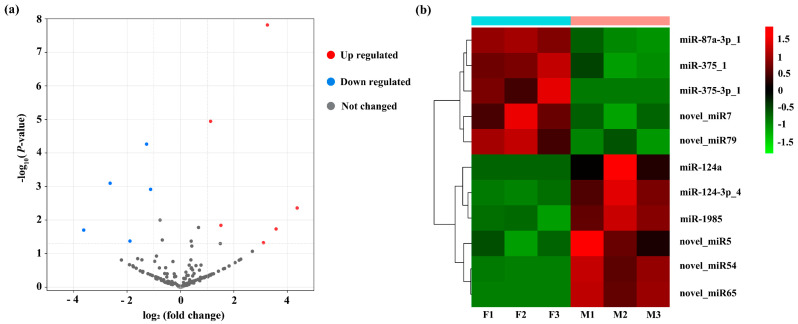
Expression profiles of miRNAs between ovary and testis of *C. farreri*. (**a**) The volcano plot exhibition of DEMs. *X*-axis, log2fold change of the miRNA expression levels between the testis and ovary. *Y*-axis, statistical significance of the miRNA expression. Red dots, upregulated miRNAs in the testis. Blue dots, upregulated miRNAs in the ovary. Grey dots, not significantly expressed miRNA between gonads. (**b**) The hierarchical clustering of miRNAs with the significant expression differences. Green or red on the heat map indicates a decrease or increase in miRNA expression levels, respectively.

By comparing the expression of miRNAs between ovary and testis, a total of 11 miRNAs were identified to be differentially expressed (Figure 4a and Table S3). Five miRNAs were significantly upregulated in the ovary, while the other six miRNAs were significantly upregulated in the testis (Figure 4b). Among the ovary-biased miRNAs, the novel_miR79 showed the highest fold change (log2 fold change = −3.62, testis versus ovary), followed by novel_miR7 (log2 fold change = −2.63) and miR-87a-3p_1 (log2 fold change = −1.89), while novel_miR65 displayed the highest fold change (log2 fold change = 4.37) among the testis-biased miRNAs, followed by miR-124a (log2 fold change = 3.58) and novel_miR5 (log2 fold change = 3.25) (Table S3).

### 3.4. Target Gene Prediction and Functional Enrichment Analysis of DEMs

**Figure 5:**
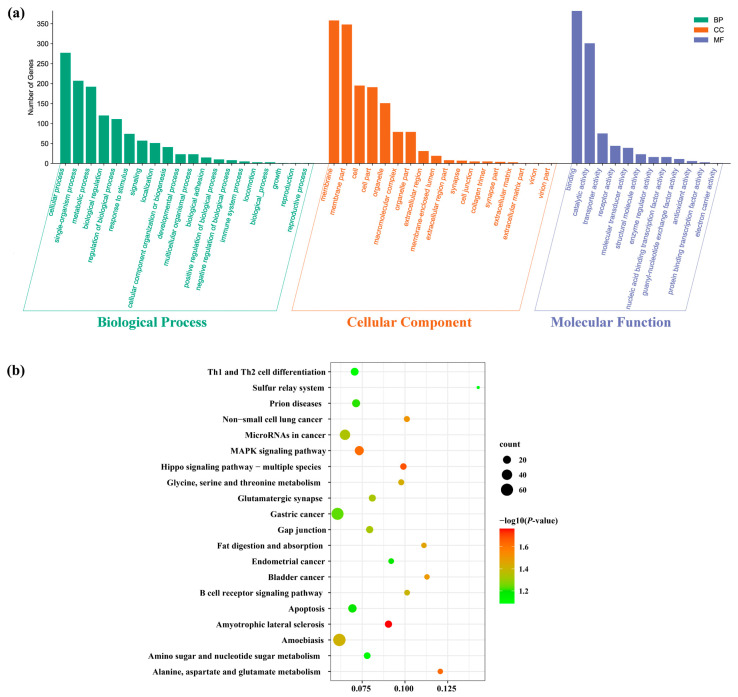
Functional analysis of the predicted target genes of DEMs. (**a**) Gene Ontology classification. *x*-axis, 50 subcategories. *y*-axis, number of genes in a specific functional cluster. (**b**) The top 20 KEGG enrichment pathways. *x*-axis, enrichment score. *y*-axis, KEGG pathway terms.

To elucidate the biological functions of the differentially expression miRNAs, the potential target genes of these miRNAs were predicted using miRanda and RNAhybrid software. Totally, 1868 putative target genes of the 11 DEMs were identified. Most of the DEMs had multiple target genes, and many genes were regulated by more than one miRNA. In order to better understand the potential regulatory role of DEMs, GO annotation and KEGG pathway enrichment analyses of their putative target genes were performed. GO annotation analysis suggested that these target genes were sorted into 50 terms under three main GO categories: biological processes, cellular components, and molecular functions. In biological processes, the targeted genes were highly enriched in the “cellular process”, “single-organism process”, and “metabolic process”. In cellular components, the targeted genes were mainly involved in the “membrane”, “membrane part”, and “cell”. In molecular function, the top three GO terms were “binding”, “catalytic activity”, and “transporter activity” (Figure 5a). The KEGG pathway analysis revealed that the target genes were annotated in 310 signaling pathways. The top 20 KEGG enriched pathways are shown in Figure 5b. Significantly, thirteen pathways were significantly enriched (*p* < 0.05) containing amyotrophic lateral sclerosis, and then the hippo-signaling pathway, followed by MAPK-signaling pathway, as well as alanine, aspartate, and glutamate metabolism.

### 3.5. Validation of DEMs Using RT-qPCR

**Figure 6:**
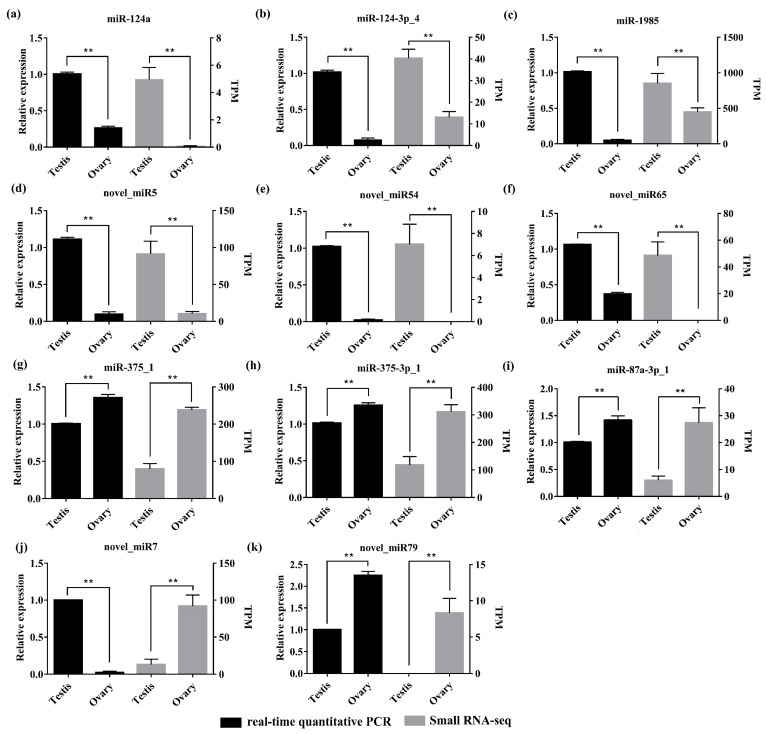
The relative expression of different miRNAs detected by RT-qPCR (left) and small RNA transcriptome data (right). (**a**)–(**f**): six testis-biased miRNAs (miR-124a, miR-124-3p_4, miR-1985, novel_miR5, novel_miR54, and novel_miR65); (**g**)–(**k**): five ovary-biased miRNAs (miR-375_1, miR-375-3p_1, miR-87a-3p_1, novel_miR7, and novel_miR79). miRNAs expression in the testis were set to “1.00” to calibrate the relative expression. Values are presented with means ± SD (*n* = 3). Asterisks (**), *p* < 0.01.

To validate the differentially expressed miRNAs identified by small RNA transcriptome data, RT-qPCR was performed on all of the11 DEMs, including six testis-biased miRNAs (cfa-miR-124a, cfa-miR-124-3p_4, cfa-miR-1985, cfa-novel_miR5, cfa-novel_miR54, and cfa-novel_miR65) and five ovary-biased miRNAs (cfa-miR-87a-3p_1, cfa-miR-375_1, cfa-miR-375-3p_1, cfa-novel_miR7, and cfa-novel_miR79). The results of RT-qPCR showed that ten of the eleven miRNAs presented the similar expression patterns compared with that detected by small RNA sequencing (Figure 6). cfa-novel_miR7 was the only exception, which was highly expressed in the ovary from the sequencing database, while the RT-qPCR analysis indicated the reverse.

### 3.6. Validation of the Interaction between DEMs and Target Genes by Dual-Luciferase Reporter Assays

**Figure 7:**
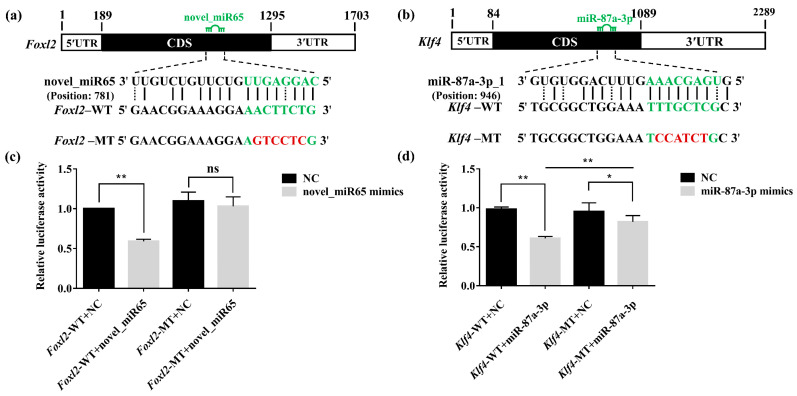
Dual-Luciferase report assay to validate the interaction between miRNAs and their targeting genes (**a**) The predicted binding sites of cfa-novel_miR65 on the CDS region of *Foxl2*; the introduced mutation sequences were in red. (**b**) The predicted binding sites of cfa-miR-87a-3p_1 on the CDS region of *Klf4*; the mutation sequences were in red. (**c**) The relative luciferase activity in cfa-novel_miR65 + *Foxl2* assay. (**d**) The relative luciferase activity in the cfa-miR-87a-3p_1 + *Klf4* assay. NC, negative control. The luciferase activity of pmirGLO-WT + NC was set to “1.00” to calibrate the relative expression. Asterisks (**), *p* < 0.01, asterisks (*), *p* < 0.05, ns, nonsignificant.

Two DEMs, cfa-novel_miR65 and cfa-miR-87a-3p_1, which were predicted to bind the coding region of a female-critical gene *Foxl2* and a male-critical gene *Klf4* respectively, were selected to further validate their interactions by dual-luciferase reporter assays [19,20]. The reporter vectors (pmirGLO-*Foxl2*-WT, pmirGLO-*Klf4*-WT, pmirGLO-*Foxl2*-MT, and pmirGLO-*Klf4*-MT) and NC mimics or miRNA mimics were co-transfected in HEK293T cells, and then, the luciferase activities were measured at 48 h after transfection. The results showed that cfa-novel_miR65 extremely downregulated the expression levels of *Foxl2* (*p* < 0.01) as compared with the control with a 40.1% reduction, but no significant difference was presented between the *Foxl2*-MT vectors and negative control (Figure 7c). cfa-miR-87a-3p_1 also extremely downregulated the expression level of *Klf4* (*p* < 0.01) by 37.5% as compared to the negative control, whereas a 13.3% downregulation existed when *Klf4*-MT vector was utilized, but a significant difference existed between the *Foxl2*-WT and *Foxl2*-MT (Figure 7d).

## 4. Discussion

MicroRNAs, as the gene expression regulators, play important roles in sex determination and differentiation of animal gonads [8,9,10,21,22,23]. To understand the roles of miRNAs in sex differentiation in scallops, we performed small RNA transcriptome sequencing of the ovary and testis from *C. farreri* juveniles with 5.0 mm shell height, a critical stage of gonadal sexual initial differentiation, identified miRNAs involving in sex-related gene regulation, and explored the miRNA–mRNA networks underlying the posttranscriptional regulation in *C. farreri* for the first time.

In the present study, most of the clean reads identified were mapped to the *C. farreri* genome (77.20% for ovaries, 73.19% for testes) (Table 1). This result was similar to that found in the small RNA libraries of *C. hongkongensis* gonads (70.63% for ovaries, 76.18% for testes) [11] and *C. gigas* gonads (72.72% for ovaries, 88.85% for testes) [13], which indicated that the quality of our small RNA libraries were satisfactory. The length distribution of the sequenced small RNAs in the ovaries and testes exhibited similar characteristics, which presented two peaks at 21–23 nt and 27–30 nt (Figure 1). The peak at 21–23 nt represented the classical size of Dicer cleavage products with the dominant size at 22 nt and followed by 23 and 21 nt, which was similar to that in previous studies [24,25,26], suggesting the conservation of miRNAs. The peak at 27–30 nt was derived from Piwi-interacting RNAs (piRNAs, 26–32 nt), which were thought to be involved in the silencing of transposons. A similar length distribution pattern was also observed in gonads of another bivalve species *C. gigas* [13], implying that piRNAs may also play important roles in sex differentiation of gonads and worth further study. U was the dominant nucleotide at the 5′ end of the known conserved miRNAs, accounting for 70% of the miRNAs. The results were consistent with other studies [27,28]. The phenomenon of nucleotide bias might be related to induce and enhance the action mechanisms of miRNA binding to the target gene.

In *C. farreri*, we obtained 75 known and 103 novel miRNAs from the ovary and testis libraries and identified six testis-biased miRNAs (cfa-miR-124a, cfa-miR-124-3p_4, cfa-miR-1985, cfa-novel_miR65, cfa-novel_miR54, and cfa-novel_miR5), as well as five ovary-biased miRNAs (cfa-miR-87a-3p_1, cfa-miR-375_1, cfa-miR-375-3p_1, cfa-novel_miR79, and cfa-novel_miR7) based on their expression profile in the testes and ovaries (Tables S4 and S5). Furthermore, we compared the expression similarities and differences of six sex-biased miRNAs (testis-biased miRNAs: miR-124a, miR-124-3p_4, and miR-1985; ovary-biased miRNAs: miR-87a-3p_1, miR-375_1, and miR-375-3p_1) between *C. farreri* and other three reported bivalves: *C. gigas*, *C. hongkongensis*, and *H. cumingii* (Table 2) [11,12,13]. miR-1985 exhibited a significant testis-biased expression in both *C. farreri* and *C. gigas*, indicating its important role in testis development. miR-375_1 presented a significant ovary-biased expression in *C. farreri* and *H. cumingii*, implying that it might involve in ovary development. However, miR-124a and miR-87a-3p_1exhibited different sex biases in different bivalve species. miR-124a was highly expressed in the testis of *C. farreri*, whereas it was identified as an ovary-biased miRNA in *C. hongkongensis*. By contrast, miR-87a-3p_1 was an ovary-biased miRNA in *C. farreri* but was mainly expressed in *H. cumingii* testis. The testis-biased miR-124-3p_4 and ovary-biased miR-375-3p_1 were identified to be DEMs only in *C. farreri*. The results indicated that most of these miRNAs may play different roles during sex differentiation and gonad development in different species.

**Figure 8:**
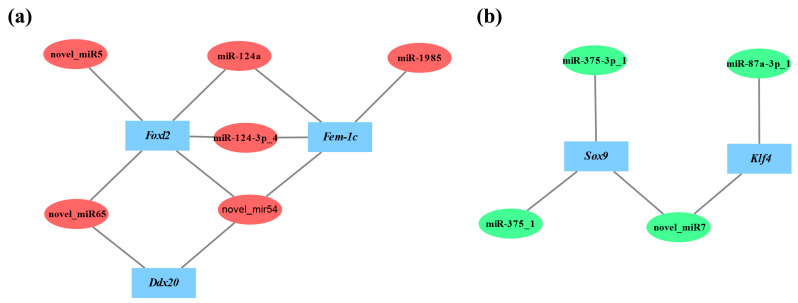
The interacting relationship between sex-related genes and DEMs. (**a**) The interactions between female-related target genes and testis-biased miRNAs. Blue rectangle, miRNA target genes. Red ellipses, testis-biased miRNAs. (**b**) The interactions between male-related target genes and ovary-biased miRNAs. Blue rectangle, miRNA target genes. Green ellipses, ovary-biased miRNAs.

To gain an insight into the potential function of DEMs, we predicted their corresponding target genes. Several of them which are important in sex determination and differentiation were identified. For example, *Sox9* (SRY-like, HMG-box-containing gene family, member 9), a testis determining gene, was targeted by miR-375-3p_1 and novel_miR7 [29,30]. *Fem-1c* (Feminization-1C), a key gene in the regulation of ovary differentiation, was targeted by miR-124a, miR-1985, and novel_miR54 [31,32,33]. While *Ddx20* (DEAD-box helicase 20), a gene required for modulating ovarian morphology and function, was targeted by novel_miR65 and novel_miR54 [34]. Each coding gene may be targeted by multiple miRNAs, and each miRNA may interact with many different target genes [35,36,37]. Therefore, miRNAs and mRNAs should be not a one-to-one relationship and might form a complex interaction network. To explore the relationships between DEMs and the corresponding sex-related target genes, we constructed the regulatory networks for female- and male-related genes, respectively (Figure 8). Based on the networks, we found the negatively correlated expression relationship between DEMs and sex-related genes. The ovary-biased DEMs always targeted those genes associated with testis development and vice versa. To further characterize these DEMs, we conducted GO enrichment and KEGG pathway analyses for those predicted target genes, which were classified into 50 GO terms (Figure 5a) and significantly enriched in 13 pathways, including the MAPK-signaling pathway, which was tightly associated with the male reproductive function [38,39], and the Hippo-signaling pathway, which was required for regulating the female reproductive system development [40,41] (Figure 5b) The results suggested that these DEMs may affect or even control sex differentiation by targeting their sex-associated target genes.

Interestingly, two sex-critical genes, *Foxl2* and *Klf4*, were identified to be targeted by DEMs in the present study. FOXL2, a member of the forkhead box family, was an evolutionarily conserved female-related gene, which was specific expressed in the ovary and mainly involved in ovarian differentiation, oogenesis, and ovarian function maintenance [42,43,44]. It is worth noting that *Foxl2* was verified to be the female sex determination gene in the goat [45]. In the bivalves, *Foxl2* had been reported to be dominantly expressed in the ovary and also considered to be a female-critical gene in *C. farreri*, *C. gigas*, *Patinopecten yessoensis*, and *Argopecten irradians* [19,46,47,48]. Several miRNAs have been discovered to regulate *Foxl2* expression. For example, miR-937 inhibit cell proliferation and metastasis in gastric cancer cells by downregulating FOXL2 [49]. miR-133b could inhibit the expression of FOXL2 mRNA and protein in COV434 cells and inhibit its migration and proliferation [50]. Meanwhile, Krüppel-like factor 4 (KLF4), a kind of zinc finger transcription factor, had also been reported to be expressed in post-meiotic germ cells of human and mouse testes and functions in testicular differentiation in mammals [51,52]. In addition, *Klf4* was revealed to be a male-critical gene in *C. farreri* based on its high expression in testis and the retardance of spermatogenesis after its knock down [20]. It was reported that miR-25 inhibition could decrease the proliferation and motility of HeLa cells and promote an increase of KLF4 level [53]. miR-9-5p downregulated *Klf4* expression and influenced the progression of hepatocellular carcinoma [54]. In the present study, *Foxl2* was predicted as a target gene of multiple male-biased miRNAs (miR-124a, miR-124-3p_4, novel_miR65, novel_miR54, and cfa-novel_miR5), while *Klf4* was predicted as a target gene of two female-biased miRNAs (miR-87a-3p_1 and novel_miR7). None of these miRNAs were reported previously to have the regulatory roles on *Foxl2* or *Klf4*, indicating that species specificity may exist.

By comparing the free energy of different miRNA–mRNA pairs, cfa-novel_miR65 and cfa-miR-87a-3p_1 with the minimum free energy that targeting *Foxl2* and *Klf4*, respectively, were selected to further validate their interactions by dual-luciferase reporter assays. The results showed that the introduction of cfa-novel_miR65 significantly decreased the luciferase activity, indicating that cfa-novel_miR65 could inhibit *Foxl2* expression by directly binding to its CDS region (Figure 7a). The repression of *Klf4* by cfa-miR-87a-3p_1 was also validated in vitro (Figure 7b). In previous research, miRNAs binding to the CDS sequence primarily resulted in translation inhibition [5,53,54], so further in vivo investigation would be followed up to demonstrate the detailed mechanisms. It was the first time that miRNAs that regulating the sex-critical genes were excavated and verified.

## 5. Conclusions

In the present study, small RNAs were sequenced from the ovary and testis of *C. farreri* juveniles at the initial sex differentiation stage. A total of 75 known miRNAs and 103 novel miRNAs were identified, of which 11, including six known miRNAs and five novel miRNAs, were differentially expressed between the sexes. The predicted target genes of DEMs related to sex differentiation were obtained by GO analysis and KEGG pathway enrichment. Furthermore, the negative regulation between cfa-novel_miR65 and *Foxl2*, as well as between cfa-miR-87a-3p_1 and *Klf4*, were validated by the dual-luciferase reporter assay. Our findings provided an important basis for the study of sex determination and differentiation mechanisms in bivalves, as well as the development of potential sex control techniques in aquaculture.

## Figures and Tables

**Table 1:** Overview of the small RNA sequencing data.

Sample	Raw Reads	Low Quality	Invalid Adapter	Poly N Reads	Short Valid Length	Clean Reads	Mapped Reads	Mapped Percentage	Q20 (%)	Q30 (%)
**F1**	30,119,160	102,088	279,083	921	394,152	29,342,916	23,187,887	79.02	99.0	96.8
**F2**	29,055,638	95,864	969,435	5444	1,406,610	26,578,285	20,211,226	76.04	98.9	96.8
**F3**	30,063,513	123,937	872,204	1424	1,350,206	27,715,742	21,220,447	76.56	98.7	96.2
**M1**	29,785,617	105,133	356,592	1123	793,287	28,529,482	22,027,953	77.21	98.9	96.5
**M2**	29,832,065	98,316	411,700	1059	2,013,570	27,307,420	18,620,412	68.19	98.9	96.7
**M3**	30,537,297	129,186	243,137	975	139,038	30,024,961	22,272,516	74.18	98.8	96.4

**Table 2:** Sex preference of six important DEMs in *C. farreri* and three reported bivalves.

Species Name	miR-124a	miR-124-3p_4	miR-1985	miR-87a-3p_1	miR-375_1	miR-375-3p_1
*C. farreri*	T	T	T	O	O	O
* C. gigas *	-	-	T	-	-	-
* C. hongkongensis *	O	-	-	-	-	-
* H. cumingii *	-	-	-	T	O	-

T, testis-biased miRNA; O, ovary-biased miRNA.

## Data Availability

The data presented in this study can be found in this article or the Supplementary Materials.

## Supplementary Materials

The following supporting information can be downloaded at https://www.mdpi.com/article/10.3390/biology11030456/s1, Table S1: Specific primers used for RT-qPCR of the miRNAs. Table S2: Specific primers for amplifying the target gene fragments. Table S3: The DEMs between testis and ovary. Table S4: Known miRNAs from ovary and testis libraries. Table S5: Novel miRNAs from ovary and testis libraries. Figure S1: Histological structure of gonad in juveniles with shell height of 5.0 mm.
