# Supplementary material for: Identification and Characterization of MicroRNAs Involving in Initial Sex Differentiation of Chlamys farreri Gonads
Source: Biology (Basel). 2022 Mar 16;11(3):456. doi: 10.3390/biology11030456 (PMC8945268; doi:10.3390/biology11030456)

Figure Legends

Figure S1. Histological structure of gonad in juvenile with shell height of 5.0 mm

In, intestine; FC, follicle cell. Arrow shows the position of follicle cells.

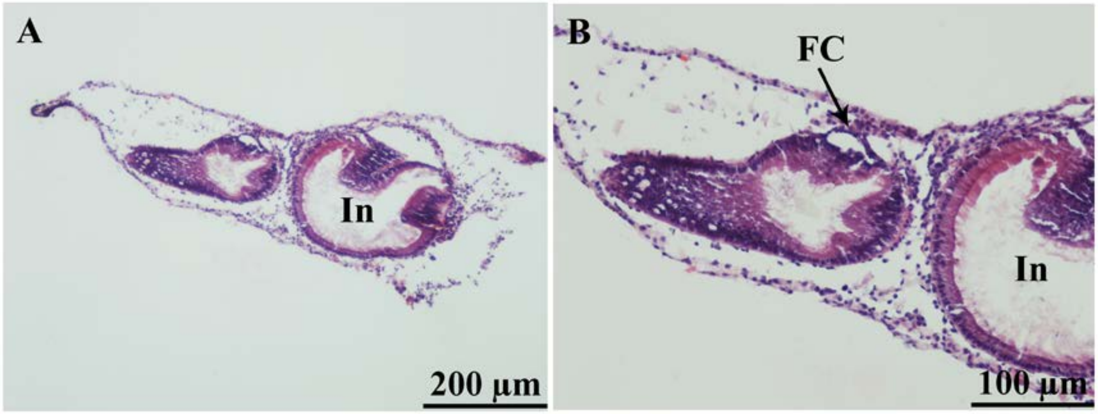

Supplement: Supplementary file 1 [file biology-11-00456-s001.zip › Fiugre S1.pdf]
