# Supplementary material for: Identification and Characterization of MicroRNAs Involving in Initial Sex Differentiation of Chlamys farreri Gonads
Source: Biology (Basel). 2022 Mar 16;11(3):456. doi: 10.3390/biology11030456 (PMC8945268; doi:10.3390/biology11030456)
Supplement: Supplementary file 1 [file biology-11-00456-s001.zip › Table S1-S3.pdf]

Identification and Characterization of MicroRNAs involving in Initial Sex Differentiation of *Chlamys farreri* Gonads

Xixi Li[1], Siyu Lin[1], Shutong Fan[1], Xiaoting Huang[1], Zhifeng Zhang[1,2,*], Zhenkui Qin[1,*]

1 Ministry of Education Key Laboratory of Marine Genetics and Breeding, College of Marine Life Sciences, Ocean University of China, Qingdao 266003, China

2 Key Laboratory of Tropical Aquatic Germplasm of Hainan Province, Sanya Oceanographic Institute, Ocean University of China, Sanya 572000, China

*** Correspondence: zzfp107@ouc.edu.cn (Z.Z.); qinzk@ouc.edu.cn (Z.Q.)**

Table S1: Specific primers used for RT-qPCR of the miRNAs

**miRNA**	**Forward primer sequence（5′-3′）**
novel_miR5	TTACCCTGTAGAACCGAGCGAGT
novel_miR7	CCCGTGATAGCTCAGTTGGTAG
novel_miR54	GATCTCTCCAAGGCCTTCGG
novel_miR65	GCAGGAGTTGTCTTGTCTGTT
novel_miR79	TGAGTAGCAGCCGGGACC
miR-87a-3p_1	GTGAGCAAAGTTTCAGGTGTG
miR-124a	TAAGGCACGCGGTGAATGCC
miR-124-3p_4	TAAGGCACGCGGTGAATGCCA
miR-375_1	TTTGTTCGTTCGGCTCGCGTTA
miR-375-3p_1	TTTGTTCGTTCGGCTCGCGTTATGTGC
miR-1985	CATTTTTATCAGTCACTGTG
5S rRNA	TATCACGTTGAAAACACCGGTTCTCG

The reverse primer was a universal primer (Takara, Dalian, China)

Table S2: Specific primers for amplifying the target gene fragments

Target gene	Primer Sequence（5′-3′）
Foxl2-WT-S	CGAGCTCAACAGTATCCGCCACAACCT
Foxl2-WT-AS	CCTCGAGAGTGGTTGTCAGCAAATAAAGG
Foxl2-MT-S	GGTGAACGGAAAGGAAGTCCTCGGACTTGGA
Foxl2-MT-AS	GAGGACTTCCTTTCCGTTCACCTCCACCTTCCCTCGGCA
Klf4-WT-S	CGAGCTCCTCCCATCTGAAAGCACACT
Klf4-WT-AS	CCTCGAGGTGGCACTGGAAAGGACGAT
Klf4-MT-S	GATGCGGCTGGAAATCCATCTGCTCTGATGAAC
Klf4-MT-AS	AGATGGATTTCCAGCCGCATCCCTTCCATGCGC

WT: wild-type; MT: mutant type; S: sense; AS: antisense; Single underline: cleavage sites of restriction enzymes; Double underline: mutant bases.

Table S3: The DEMs between testis and ovar

miRNAs	Testis (TPM)	Ovary (TPM)	Log_2(fold change)	*p*-value	mature miRNAs sequence
miR-124a	71.1125516840033	5.93560650481348	3.5826368662642	0.018463927	UAAGGCACGCGGUGAAUGCC
miR-124-3p_4	766.810265851032	268.214396432376	1.51548297544166	0.01440008	UAAGGCACGCGGUGAAUGCCA
miR-1985	17491.3396364478	8001.10215998119	1.12837013590651	1.14E-05	UGCCAUUUUUATCAGUCACUGUG
miR-375_1	1648.50526430289	3968.69846821903	-1.26750746005321	5.45E-05	UUUGUUCGUUCGGCUCGCGUUA
miR-375-3p_1	2433.17741785888	5279.77768604446	-1.11763566391377	0.001216834	UUUGUUCGUUCGGCUCGCGUUAU
miR-87a-3p_1	119.888137867487	444.997115730411	-1.8921070644632	0.042434472	GUGAGCAAAGUUUCAGGUGUG
novel_miR5	1905.42516714067	199.343983935073	3.25678097791346	1.53E-08	UUACCCUGUAGAACCGAGCGAGU
novel_miR54	74.4307965107132	8.63342185017998	3.10789528588565	0.047379671	GAUCUCUCCAAGGCCUUCGG
novel_miR65	418.93997433732	20.2413341945236	4.37136725698746	0.004382968	CAGGAGUUGUCUUGUCUGUU
novel_miR7	238.380178479512	1480.41700967009	-2.63466743255438	0.000801096	CCCGUGAUAGCUCAGUUGGUAG
novel_miR79	8.420054401	103.75193373645	-3.62316486105696	0.019962792	UGAGUAGCAGCCGGGACCGCCCU
